# Price and Availability of Sugar-Free, Sugar-Reduced and Low Glycemic Index Cereal Products in Northwestern México

**DOI:** 10.3390/ijerph14121591

**Published:** 2017-12-18

**Authors:** Jesús G. Arámburo-Gálvez, Noé Ontiveros, Marcela J. Vergara-Jiménez, Dalia Magaña-Ordorica, Martina H. Gracia-Valenzuela, Francisco Cabrera-Chávez

**Affiliations:** 1 Nutrition Sciences Academic Unit, University of Sinaloa, Av. Cedros and Sauces Street, Los Fresnos, 80019 Culiacán, Sinaloa, Mexico; gilberto.aramburo.g@gmail.com (J.G.A.-G.); mjvergara@uas.edu.mx (M.J.V.-J.); dmagana@uas.edu.mx (D.M.-O.); 2 Instituto Tecnológico del Valle del Yaqui, Block 611, 82276 Bácum, Valle del Yaqui, Sonora, Mexico; hgracia@itvalledelyaqui.edu.mx

**Keywords:** cereal-based foods, sugar-free, sugar-reduced

## Abstract

Sugar-free (SF), sugar-reduced (SR), or low-glycemic-index (low GI) cereal products could be helpful for the dietary treatment of disorders related to glucose homeostasis. However, access and economic aspects are barriers that could hamper their consumption. Thus, the availability and price of such cereal products were evaluated in Northwestern México. The products were categorized in 10 groups. The data were collected in five cities by store visitation (from November 2015 to April 2016). The availability in specialized stores and supermarkets was expressed as availability rates based on the total number of products. The price of the SF, SR, and low GI products were compared with their conventional counterparts. Availability rates were higher in supermarkets than in specialized stores by product numbers (14.29% versus 3.76%, respectively; *p* < 0.001) and by product categories (53.57% versus 26.92%, respectively; *p* < 0.001). Five categories of products labeled as SF, SR, and low GI (oats, cookies and crackers, flours, snacks, and tostadas/totopos) had higher prices than their conventional counterparts (*p* < 0.05). In conclusion, in Northwestern Mexico, the availability of SF, SR, and low GI cereal-based foods is relatively low, and these foods are more expensive than their conventional counterparts.

## 1. Introduction

The restriction of sugar and cereal-based foods is recommended for the dietary treatment of disorders related to glucose metabolism such as obesity and type 2 diabetes [1,2]. The general perception is that cereal consumption increase glucose blood levels, but this depends on the nature of the cereal’s carbohydrates [3]. In this context, the food industry offers special cereal-based foods that meet the requirements to comply with special diets such as the sugar-free and/or low-glycemic-index (low GI) diets. The availability and price of these special cereal-based foods remains uncertain in most countries, but the first published reports about this topic highlighted that these products could have limited availability [4]. Contrary to the price and availability of sugar-free (SF), sugar-reduced (SR), and low GI cereal-based products, the price and availability of other cereal-based foods for special dietary uses, such as gluten-free products, have been widely documented in many countries outside Latin America [5,6,7,8]. Thus, the aim of this study was to evaluate the availability and price of cereal-based products labeled as SF, SR, and low GI in five cities from Northwestern Mexico.

## 2. Materials and Methods

Cereal-based products from a market basket published by National Council for the Evaluation of Social Development Policy [9] were categorized in 10 groups: oats, breakfast cereals, cookies and crackers, granola, flours, bread, pasta, snacks, tostadas/totopos, and tortillas. All these products can be re-formulated or processed to be labeled as SF, SR, or low GI and have conventional counterparts. The data were collected by store visitation. Specialized stores were selected (by convenience) from the National Statistical Directory of Economic Units published by INEGI [10]. Mainstream supermarkets located in five cities from Northwestern Mexico (Culiacán, Hermosillo, La Paz, Mexicali, and Tijuana) were also included in the study. The availability rate of SF, SR, and low GI products was individually calculated per supermarket or specialized store. Calculations were carried out according to the following equations for each store and was defined as follows [7]:

Based on the total number of different products,

$$AR_{P}=(\frac{NP}{TP})\times 100.$$

Based on the number of food categories (10 categories),

$$AR_{C}=(\frac{NC}{TC})\times 100.$$

*AR_P_*: availability rate by product.
*NP*: number of different SF, SR, and low GI products available per supermarket or specialized store.
*TP*: total of different SF, SR, and low GI products available in Northwestern México (Σ of the different versions of SF, SR, and low GI products available in all the stores visited).
*AR_C_*: availability rate by category.
*NC*: number of categories with at least one SF, SR, or low GI food product available in a single store.
*TC*: total of food product categories (*TC* = 10 in this study).

The data points were plotted individually and mean ± SD calculated. Availability rates of SF, SR, or low GI products were individually calculated based on the total number of products available (including supermarkets and specialized stores).

The price of the six most expensive and the six cheapest products by categories were recorded. The average price of all 12 items corresponding to each food category was calculated for each supermarket and specialized store and used for statistical comparisons. This approach “ensure that the cost of standard versions was not excessively influenced by the wider availability of ‘quality’ or ‘basic’ products” [6]. The price was expressed as MX$/100 g of product. For statistical comparisons, Mann–Whitney U tests were carried out using PAWS statistics version 22.0.0.0 (SPSS Inc., Chicago, IL, USA). For price comparisons, the average store price of SF, SR, and low GI foods were compared to the average store price of regular foods, based on the average of the 6 most expensive and the 6 least expensive items within each store. A minimum of four establishments contributing data to the price was considered as an arbitrary cut-off point to perform the statistical analysis.

## 3. Results

### 3.1. Availability

A total of 13 specialized stores and 29 supermarkets were visited in five cities. There were 131 different SF, SR, and low GI products available. SF products had more availability (74.81%; *n* = 98) than the SR (16.03%; *n* = 21) or low GI (9.16%; *n* = 12) ones. The product category with the highest number of different versions available was cookies and crackers (*n* = 59), while the category ‘tortilla’ had only one product available. The other categories had 4–15 products available.

Of the 131 products available, 23 were endorsed by at least one of the following organizations: Mexican Federation of Diabetes, Mexican Association of Diabetes, Mexican Association of Nutrition and Diabetes, Spanish Diabetes Society Foundation, and the Glycemic Index Foundation. The most commonly endorsed products were cookies (*n* = 15) labeled as SF, followed by bread and snacks (*n* = 2 each), pasta, flour, granola, and breakfast cereal (*n* = 1 each). Among the low GI products, 5 out of 12 declared the precise value of glycemic index.

**Figure 1:**
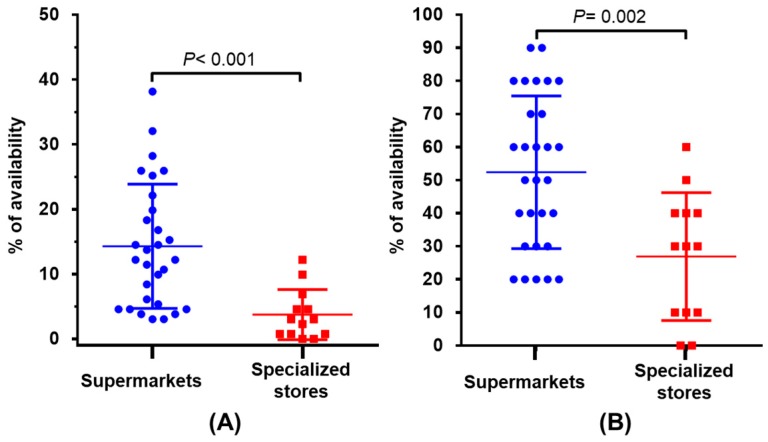
Availability of cereal-based products labeled as sugar-free (SF), sugar-reduced (SR), or low glycemic index (low GI) in supermarkets and specialized stores. (**A**) Availability (*Ar_p_*) based on the total number of products (*n* = 131); (**B**) Availability (*AR_c_*) considering the total of categories (10).

Figure 1 shows the availability of cereal-based products labeled as SF, SR, or low GI. When the analysis was performed considering the total number of products available (Part A), most supermarkets and specialized stores had <15.0% of availability. In this context, the mean values of availability were 14.29% and 3.76% for supermarkets and specialized stores, respectively (*p* < 0.001). When the analysis of availability was carried out based on the food categories (Part B), the specialized stores had lower availability than supermarkets (26.92% vs. 53.57%, respectively) (*p* = 0.002). In this last analysis, the highest availability rates were 90.00% and 60.00% in supermarkets and specialized stores, respectively. Notably, 48.00% of the supermarkets had availability rates ≥60.00% considering the 10 categories (average across the 10 categories). 

### 3.2. Price

Table 1 shows the price comparisons between SF, SR, low GI products, and conventional products. Five categories were significantly more expensive than their conventional counterparts (*p* < 0.05). There was only one specialized product called tortilla, which was labeled as low GI, and its price was 3.44 times higher than the national regularized price of maize tortilla. The bread category included mostly buns labeled as SF and SR (*n* = 10). Price comparison between regular and non-regular bread was not significant (*p* = 0.334). However, price comparison between white bread labeled as low GI (*n* = 5) and the conventional white bread (*n* = 28) was significant (*p* < 0.001).

## 4. Discussion

In this study, we evaluated the availability and price of foods labeled as SF, SR, and low GI. SF and SR cereal-based products were more available than the low GI ones. From a technological point of view, the formulation SF and SR cereal-based products represents a less complicated technological challenge than the elaboration of low GI cereal products. For instance, products containing sugar substitutes instead of sugar can be labeled as SF or SR, but the development of low GI cereal products requires the substitution of native cereal carbohydrates by fiber, resistant starches or slow absorption carbohydrates [11,12,13,14]. Another reason that could explain the limited availability of low GI cereal products is the fact that the declaration of low GI is not mandatory by Mexican legislations [15].

The availability of SF, SR, and low GI products was higher in supermarkets than in specialized stores. Similarly, Patton et al. [16] reported that independent stores and small markets had lower availability of healthy alternative foods than supermarkets. The fact that only two 2 out of 29 supermarkets had an availability of 90.00% (9 out of 10 categories) indicates that people trying to cover a market basket should visit more than one establishment to complete it. Furthermore, based on the analysis by the number of products, there will be a poor variety of products labeled as SF, SR, or low GI. Accordingly, a recent study showed that the general perception of individuals with diabetes of a low socioeconomic status is that specialized food products have limited availability [4].

The data highlight that some cereal-based foods are cheaper than their SF, SR, and low GI counterpart. In fact, 5 out of 10 SF, SR, and low GI food categories were significantly more expensive than their conventional counterparts. The perception is that the high price of specialized products limit their daily consumption by people that requires SF, SR, and low GI food products [16]. Our study partially supports such a perception since a half of the food categories of SF, SR, and low GI products had a significantly higher price than their conventional counterparts. Moreover, the trend was similar in all the categories.

## 5. Conclusions

SF, SR, and low GI cereal-based products have limited availability and tend to be more expensive than their regular counterparts in Northwestern Mexico. These access and economic aspects could make it difficult to comply with sugar restricted diets, which are recommended in the dietary treatment of conditions related to glucose metabolism.

## Figures and Tables

**Table 1:** Price comparison between cereal products labeled as SF, SR, or low GI and their regular counterparts.

Category of Food	Mean * (SD) (Number of Establishments Contributing Data to the Price)		*p*
	SF, SR, and low GI	Regular	
Oats	11.4 (1.8) (21)	7.3 (2.2) (29)	<0.05
Breakfast Cereal	14.7 (11.6) (20)	10.8 (1.7) (28)	0.151
Cookies and crackers	20.5 (10.0) (36)	12.5 (3.9) (29)	<0.05
Granola	11.3 (2.4) (24)	10.0 (2.1) (29)	0.057
Flour	15.6 (13.04) (14)	4.7 (1.0) (29)	0.008
Bread	10.9 (2.5) (29)	10.1 (3.9) (27)	0.334
Pasta	9.1 (8.7) (19)	5.9 (3.1) (28)	0.144
Snack	35.6 (14.8) (19)	20.3 (6.0) (29)	<0.05
Tostadas/totopos	27.5 (11.0) (6)	7.9 (2.0) (25)	0.007
Tortilla	5.0 (0) (1)	1.45 ^#^	ND ^##^

* Price in Mexican pesos per 100 g of product (1 USD = 17.7 MX$). Average store price and SD, based on the average of the six most expensive and the six least expensive items. ^#^ Average of national price of maize tortilla. ^##^ Not enough data for the analysis.
